# The Potential Usefulness of ChatGPT in Oral and Maxillofacial Radiology

**DOI:** 10.7759/cureus.42133

**Published:** 2023-07-19

**Authors:** Jyoti Mago, Manoj Sharma

**Affiliations:** 1 Oral and Maxillofacial Radiology, University of Nevada, Las Vegas (UNLV), Las Vegas, USA; 2 Public Health, University of Nevada, Las Vegas (UNLV), Las Vegas, USA

**Keywords:** radiographic features, pathology, oral and maxillofacial radiology, chatgpt, open-ai

## Abstract

Aim

This study aimed to evaluate the potential usefulness of Chat Generated Pre-Trained Transformer-3 (ChatGPT-3) in oral and maxillofacial radiology for report writing by identifying radiographic anatomical landmarks and learning about oral and maxillofacial pathologies and their radiographic features. The study also aimed to evaluate the performance of ChatGPT-3 and its usage in oral and maxillofacial radiology training.

Materials and methods

A questionnaire consisting of 80 questions was queried on the OpenAI app ChatGPT-3. The questions were stratified based on three categories. The categorization was based on random anatomical landmarks, oral and maxillofacial pathologies, and the radiographic features of some of these pathologies. One oral and maxillofacial radiologist evaluated queries that were answered by the ChatGPT-3 model and rated them on a 4-point, modified Likert scale. The post-survey analysis for the performance of ChatGPT-3 was based on the Strengths, Weaknesses, Opportunities, and Threats (SWOT) analysis, its application in oral and maxillofacial radiology training, and its recommended use.

Results

In order of efficiency, Chat GPT-3 gave 100% accuracy in describing radiographic landmarks. However, the content of the oral and maxillofacial pathologies was limited to major or characteristic radiographic features. The mean scores for the queries related to the anatomic landmarks, oral and maxillofacial pathologies, and radiographic features of the oral and maxillofacial pathologies were 3.94, 3.85, and 3.96, respectively. However, the median and mode scores were 4 and were similar to all categories. The data for the oral and maxillofacial pathologies when the questions were not specifically included in the format of the introduction of the pathology, causes, symptoms, and treatment. Out of two abbreviations, one was not answered correctly.

Conclusion

The study showed that ChatGPT-3 is efficient in describing the pathology, characteristic radiographic features, and describing anatomical landmarks. ChatGPT-3 can be used as an adjunct when an oral radiologist needs additional information on any pathology, however, it cannot be the mainstay for reference. ChatGPT-3 is less detail-oriented, and the data has a risk of infodemics and the possibility of medical errors. However, Chat GPT-3 can be an excellent tool in helping the community in increasing the knowledge and awareness of various pathologies and decreasing the anxiety of the patients while dental healthcare professionals formulate an appropriate treatment plan.

## Introduction

With the advent of technology, artificial intelligence is emerging to be and is currently among the most researched technologies. Alan Turing, in 1950, suggested the Turing test, which is a test to compare if a machine can accomplish intelligence at the human level [1]. Five years later, in 1955, the terminology AI was first suggested in a two-month workshop led by McCarthy J et al. [2]. Machine learning (ML) algorithms; and evidence-based dentistry both aim at analyzing the current advancements in healthcare and precision medicine to minimize human error [3]. Compared to evidence-based dentistry, machine learning can achieve its aims faster due to the method of how the data are collected [3]. However, machine learning requires a lot of data collection, which may be biased [3].

Chat Generated Pre-Trained Transformer-3 (ChatGPT-3) is an OpenAI model and a powerful member of artificial intelligence-generated content (AIGC) [4]. GPT-1 model was released in 2018, and its modification ChatGPT-3 (OpenAI; San Francisco, CA), which is a large language model (LLM), was developed to leverage its use in November 2022, and is largely being studied to guide clinical decision-making, and as a guide to education [5].

These artificial intelligence-based software applications can provide text-based responses by using natural language [6]. The ChatGPT-3 has an added advantage over the previously designed GPT models in that on the basis of advanced modeling, it has the ability to respond to multiple languages [7,8].

A newer version of this technology, GPT-4 has been introduced in March 2023 but is currently not available for public use [5]. ChatGPT-4 has been trained using both supervised and unsupervised learning methods on a huge amount of data from the Internet and reinforcement learning with human feedback [5]. At times, it is not feasible for patients to ask the concerns of referring doctors where this technology may be of help in answering their questions.

In academics, ChatGPT got varied responses, based on the collection of the data, and highlights the debate regarding the risks vs. benefits of using open AI models [9-11]. Therefore, the current study uses the ChatGPT-3 technology, and the aim was to evaluate the potential usefulness of ChatGPT-3 in oral and maxillofacial radiology for report writing by analyzing its accuracy and to further analyze its usage and experience by post-survey by an oral and maxillofacial radiologist.

## Materials and methods

An experienced oral and maxillofacial radiologist queried a questionnaire consisting of 80 questions to the open-AI ChatGPT-3 Model application; Chaton (AIBY Inc., Miami, Florida, version 1.13). The questionnaire was validated for face and content validity by the researchers themselves. The Flesch-Kincaid reading grade level of the questionnaire was 15.6 implying that it required at least college-level preparation to read the questionnaire. The random questions were categorized based on anatomical landmarks of the head and neck region and comprised 19 questions, 31 queries from a few of the oral and maxillofacial pathologies, and 30 queries from the radiographic features of some of these pathologies, and out of all queries, randomly two abbreviations were queried one from pathology section and other from the anatomical landmark. The list of queries has been supplied in the supplemental material (Appendices) and a few of them are in Table 1.

**Table 1:** Sample questions for oral and maxillofacial report writing

Sr. No.	Sample Question	ChatGPT Likert Scale
1.	What is Dense Bone Island?	4
2.	Most common odontogenic cyst?	4
3.	What are petroclinoid ligaments?	4
4.	Mastoid foramen location?	4
5.	What is zygomaticotemporal suture?	4
6.	Superior orbital fissure location?	4
7.	What is Gardner’s syndrome?	4
8.	What is cleft lip and cleft palate?	4
9.	What is hemifacial microsomia?	4
10.	What is mandibulofacial dysostosis?	4
11.	What is cleidocranial dysplasia?	4
12.	What is hemifacial hyperplasia?	4
13.	What is segmental odontomaxillary dysplasia?	4
14.	What is condylar hyperplasia?	4
15.	What is condylar hypoplasia?	4
16.	What is a bifid condyle?	4
17.	Radiographic features of condylar hyperplasia?	4
18.	Radiographic features of Goldenhar syndrome?	4
19.	What is OKC?	1
20.	Ameloblastoma	3

The evaluator in a time frame of one month queried the ChatGPT-3 application and evaluated them using a 4-point modified Likert scale, which was adapted from Muttanahaly et al. [12]: (4) The application responded with adequate information such as mentioning the characteristic features or explaining the basic pathophysiology; (3) The application responded, however, did not provide adequate information. For instance, a one-line answer which does not describe any characteristic feature (2); The application did not know the response to the question; (1) The application led to error messages.

The outcomes of the retrieved queries were recorded on a Microsoft Excel sheet (Microsoft Corporation, Redmond, WA), and mean, median, and mode values were recorded using a calculator.

The post-survey analysis, which includes strength, weakness, opportunities, and threats (SWOT) analysis [13] was done to evaluate the aforementioned parameters. The additional analysis also included whether ChatGPT is useful in oral and maxillofacial radiology training, and its possible recommendations for use.

## Results

An oral and maxillofacial radiologist queried the questions regarding: (1) anatomical landmarks, (2) oral and maxillofacial pathologies, and (3) radiographic features of oral and maxillofacial pathologies. The answers were recorded, and a comparative analysis was done.

**Figure 1:**
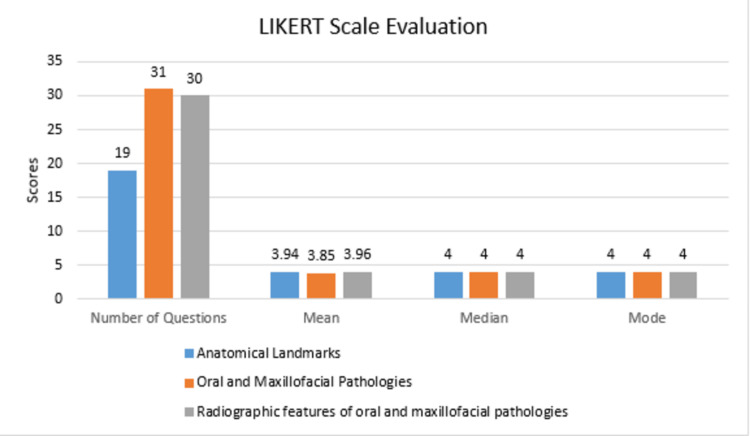
Descriptive analysis for various queries on the ChatGPT

Out of 80 questions, 19 were related to anatomical landmarks, 31 were related to oral and maxillofacial pathologies, and 30 were related to radiographic features of oral and maxillofacial pathologies. Our results indicated that of all the categories the mean score was least for the queries related to the oral and maxillofacial pathologies and the value was 3.85. The mean score for the queries related to the anatomical landmarks and radiographic features for oral and maxillofacial pathologies were 3.94 and 3.96, respectively (Figure 1).

**Figure 2:**
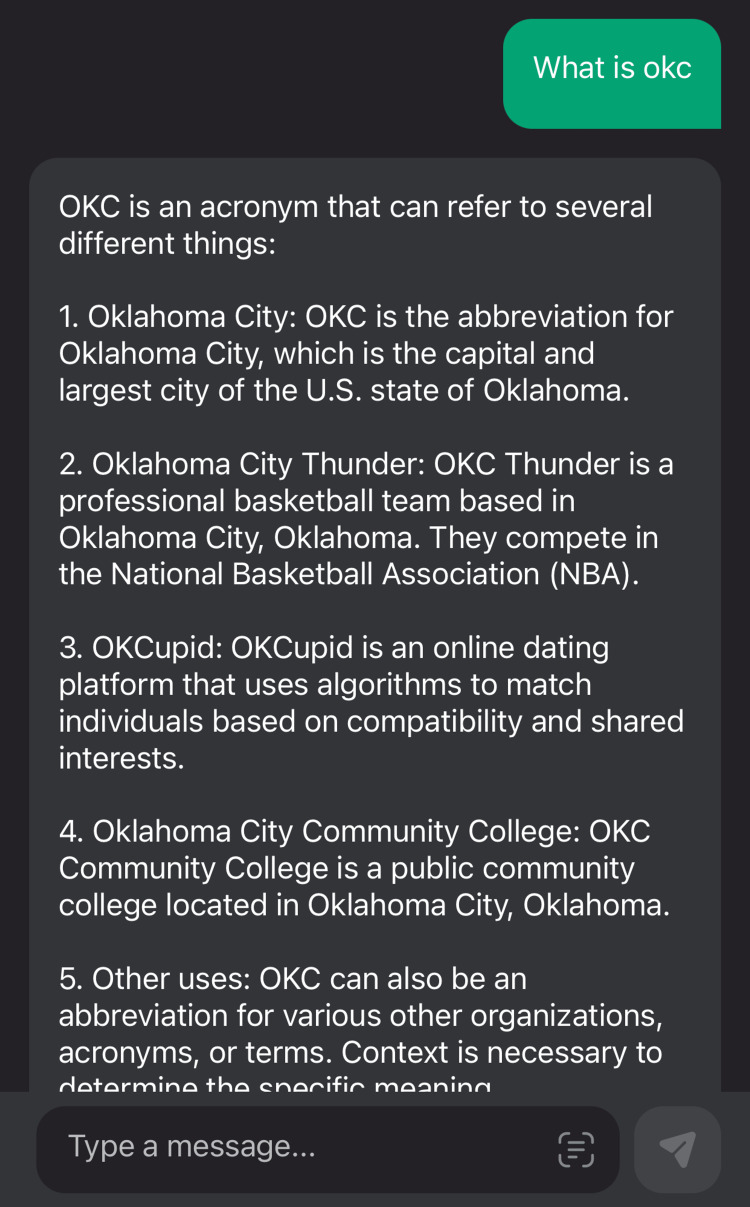
ChatGPT for the score value one due to an error message while using an abbreviation

**Figure 3:**
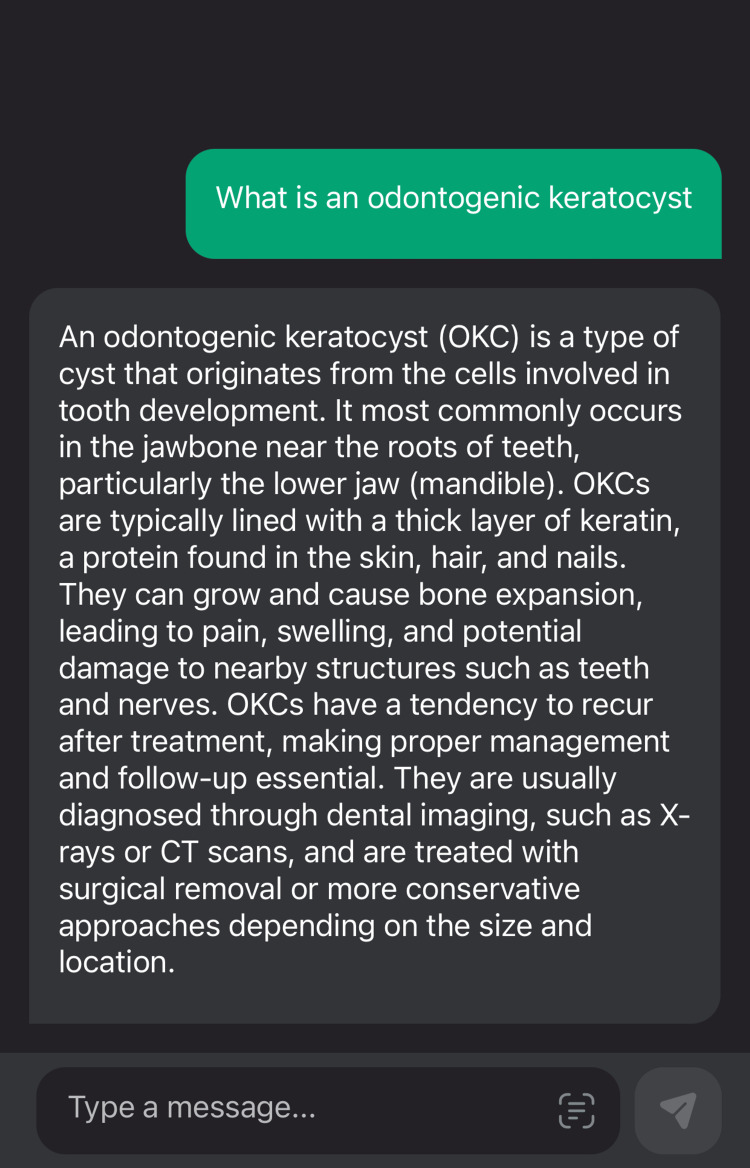
An example of a score value 4 by using a general query that was answered in the format of an introduction of the pathology, causes, symptoms, and treatment

The comparison of the same pathology when queried as an abbreviation and as a full form in Figure 2 and Figure 3, respectively, is below.

**Figure 4:**
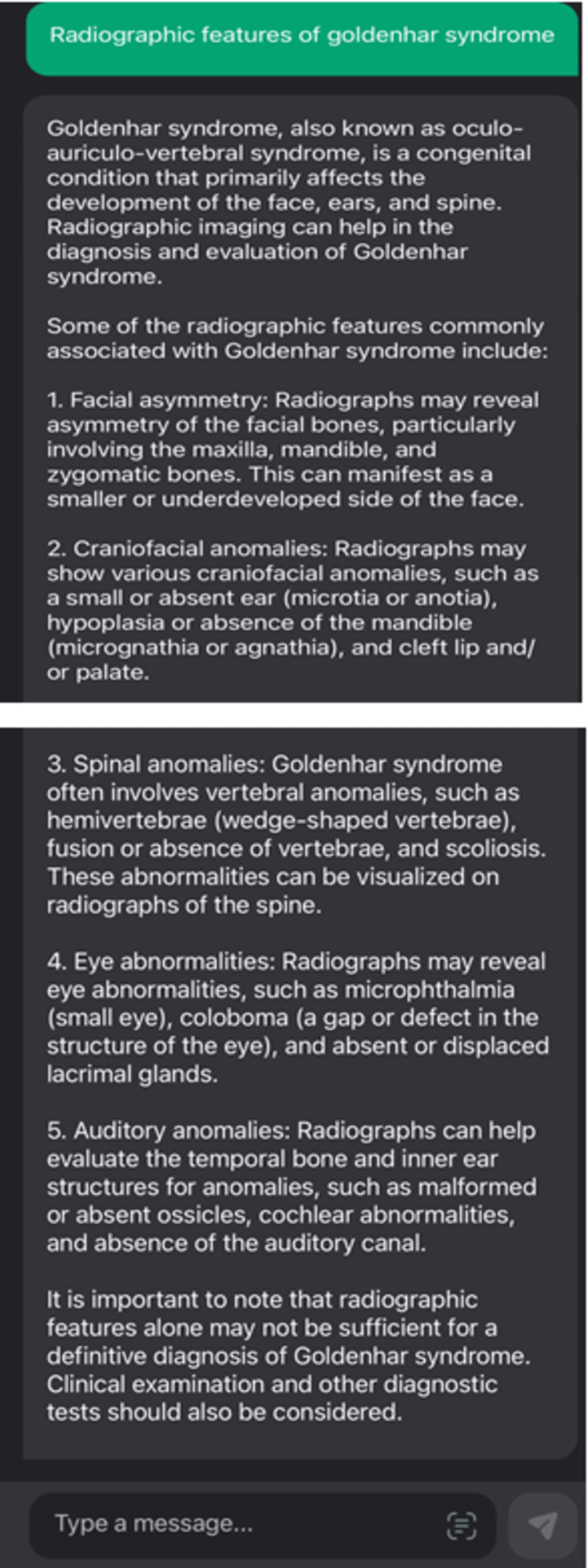
An example with a score value of 4 when ChatGPT was queried upon a specific question

**Figure 5:**
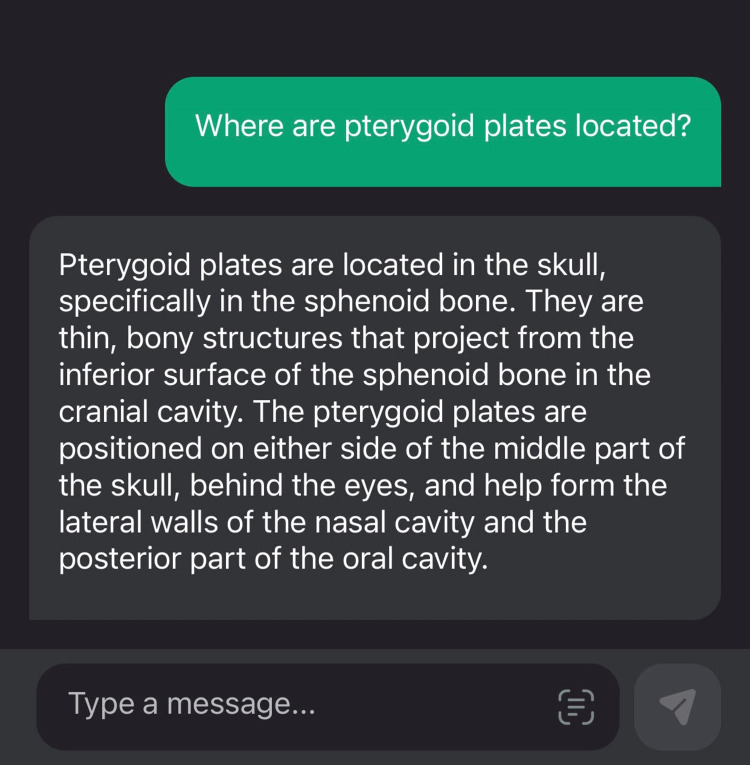
An example of a score value of 3 when ChatGPT was queried for an anatomical landmark

The sample responses for the queries of various other Likert scores are given in Figures 4-5.

Post-survey analysis

The greatest strength of ChatGPT-3 in oral and maxillofacial radiology was its ease of use and portability, as it is easy to carry than a textbook. This technology is an easy way to find the characteristic features of rare and common pathologies. The other implication could be an increase in oral healthcare education and building knowledge among patients and the community, as it may prepare the patients for the treatment plan that a dental healthcare professional formulated for them. The chief weaknesses were its reduced accuracy in answering the abbreviation. Sometimes, the data was not very specific. Additionally, further larger studies are required to rule out the possibility of infodemics. The major threats include ethical concerns, copyright issues, and legal and regulatory issues, which were also mentioned by many researchers [14-19]. This AI-powered modality has a lot of potential when used in association with proper healthcare-trained professionals to improve patient care and outcomes. This modality needs to be further developed to leverage its usage in clinical settings.

The use of ChatGPT-3 in oral and maxillofacial radiology training is not advisable because of its slight narration of the topic, possible risk of infodemics, and ethical and copyright concerns [19]. Its use is not very advantageous in radiology training because of the limited information.

## Discussion

As artificial intelligence is advancing, more models, such as open AI, are becoming more prevalent. These language-based models have the ability to interact with users via text messages [20]. Artificial intelligence-based chatbots have paved their way into various fields, such as investment, education, and customer service, and are now being in research for use in the field of healthcare as well [21-23].

Our study showed that due to text-based technology, ChatGPT-3 is easy to use. The use is handy on a mobile application and easier to carry than a textbook. However, the data are plagiarized, and this was supported by many editorials such as Science, Nature, and the Lancet editorials, which disapproved of ChatGPT as an author [24-26]. Our study revealed that even though the data when used for oral and maxillofacial pathologies and their radiographic features, it only highlights the major characteristic feature. The data do not appear to be detail-oriented. Additionally, one of our study results was that abbreviations are not taken well, for instance, in our case, where OKC, which is an abbreviation of odontogenic keratocyst, was answered incorrectly by the ChatGPt-3 model. Gilson et al. also mentioned in their study that ChatGPT was initially trained on a corpus with the data processing on or before 2021, so it does not have updated data [27]. Hence, there is a huge probability of infodemics using the old data [28].

One of the highlighted findings was that in order to get an accurate result for the queries the questions should be very specific. This application can be a good supplement for additional information and can be a good side reference for rare diseases. Since its usage by healthcare professionals is very specific and task-oriented, it may not be a useful tool for specialists or other dental healthcare professionals. This research also emphasizes the use of textbooks as a standard learning tool than the use of ChatGPT-3. Antaki et al. in 2023 when conducted a study to evaluate the use of the ChatGPT in the ophthalmology examination found that ChatGPT performance was equivalent to an average first-year resident [29]. Bhayana et al. in 2023 also suggested the possible use of this technology in radiology training to understand the basic concepts due to its successful performance in answering the queries related to clinical management [30]. Nevertheless, ChatGPT-3 is a great tool for educating the public and creating knowledge and awareness among them.

As the GPT-3 model is an AI Language model, it is not designed to read images. Hence, oral and maxillofacial radiologists need to describe the entity or pathology in detail to get leverage from this technology. However, there are other models that have the capacity to analyze the image and extract information from the same, such as ChatGPT-4, which is not available currently for public use [5]. The ChatGPT-3 model is more of a descriptive model, which limits its use in radiology. However, the use of such applications in healthcare settings can lead to the possibility of errors and medico-legal issues [31]. However, researchers such as Borji et al. also highlighted the ability of a ChatGPT in generating inaccurate results, cybersecurity concerns, and ethical and social implications [32].

The limitation of the study includes that this is a small study that queried only anatomical landmarks and features of pathologies and their radiographic analysis by a single evaluator. Larger studies with varied topics should be done to evaluate the usefulness of this technology.

The present research recommends conducting analogous, multi-institutional research on a wider scale with a wider range of questions. In a nutshell, ChatGPT-3 has a promising future as an open AI-powered model. However, newer models, such as GPT-4, can be a possible future in providing differentials, as they can read the images and itself make a robust tool in the field of medicine and dentistry. This study also highlights the human touch to achieve appropriate and acceptable levels of diagnosis due to misleading clinical scenarios, and to reduce medicolegal errors. Rao et al., in 2023, evaluated the capacity of ChatGPT-3 as an adjunct in decision-making in clinical radiology in breast pain, and breast cancer screening, and found potential in improving the clinical workflow with its usage [33]. However, ChatGPT-3 can be used in oral and maxillofacial radiology training as an educational tool, such as creating a questionnaire, if it is developed more and used by licensed healthcare professionals.

## Conclusions

ChatGPT is efficient and nearly accurate in describing the pathology, characteristic radiographic features, and describing anatomical landmarks. ChatGPT can be used as an adjunct when an oral radiologist needs additional information on any pathology or to identify a landmark, however, cannot be the mainstay for reference. This technology is a good and handy adjunct to the oral and maxillofacial radiologist and a great tool in educating and creating awareness among the public/the community about the disease process.

## Appendices

**Table 2:** List of queries for oral and maxillofacial report writing

Sr. No.	Sample Question	ChatGPT Likert Scale
1.	What is Dense Bone Island?	4
2.	Most common odontogenic cyst?	4
3.	What are petroclinoid ligaments?	4
4.	Mastoid foramen location?	4
5.	What is zygomaticotemporal suture?	4
6.	Superior orbital fissure location?	4
7.	What is Gardner’s syndrome?	4
8.	What is cleft lip and cleft palate?	4
9.	What is hemifacial microsomia?	4
10.	What is mandibulofacial dysostosis?	4
11.	What is cleidocranial dysplasia?	4
12.	What is hemifacial hyperplasia?	4
13.	What is segmental odontomaxillary dysplasia?	4
14.	What is condylar hyperplasia?	4
15.	What is condylar hypoplasia?	4
16.	What is a bifid condyle?	4
17.	Radiographic features of condylar hyperplasia?	4
18.	Radiographic features of Goldenhar syndrome?	4
19.	What is OKC?	1
20.	Ameloblastoma	3
21.	What is an odontogenic keratocyst?	4
22.	Where are pterygoid plates located?	3
23.	What are tonsilloliths?	4
24.	What are phleboliths?	4
25.	Radiographic features of simple bone cysts?	3
26.	Radiographic features of amelogenesis Imperfecta?	4
27.	Radiographic features of dentin dysplasia?	4
28.	Radiographic features of dentinogenesis Imperfecta?	4
29.	Taurodontism	4
30.	Radiographic features of brown tumor of hyperparathyroidism	4
31.	Radiographic features of systemic sclerosis in the oral cavity	4
32.	Radiographic appearance of odontogenic myxoma	4
33.	Radiographic features of malignancy	4
34.	Radiographic appearance of anterior clinoid processes	4
35.	Radiographic appearance of posterior clinoid processes	4
36.	What is lamina papyracea?	4
37.	Location of crista galli?	4
38.	What is silent sinus syndrome	4
39.	Radiographic features of silent sinus syndrome	4
40.	Radiographic features of fibrous dysplasia	4
41.	Radiographic features of osseous dysplasia	4
42.	Radiographic features of cementoblastoma	4
43.	Radiographic features of cemento-ossifying fibroma	4
44.	Radiographic features of Pierre Robin sequence	4
45.	What is Pierre Robin sequence?	4
46.	What is IAN?	4
47.	What is hypercementosis?	4
48.	Radiographic appearance of hypercementosis	4
49.	What is concha bullosa?	4
50.	Radiographic appearance of mucocele	4
51.	What are antroliths?	4
52.	Location of trochlear calcifications	4
53.	What is coronoid hyperplasia?	4
54.	Radiographic appearance of coronoid hyperplasia	4
55.	What are arachnoid granulations?	4
56.	Radiographic appearance of arachnoid granulations	4
57.	What is calcinosis cutis?	4
58.	What is Down syndrome?	4
59.	Radiographic appearance of Down syndrome	4
60.	What is hamulus?	4
61.	Location of the posterior superior alveolar artery	4
62.	Location of the greater palatine foramen	4
63.	Location of anterior superior palatal alveolar artery?	4
64.	What is sinusitis	4
65.	What is mucositis?	4
66.	What is Paget’s disease?	4
67.	Radiographic appearance of Paget’s disease	4
68.	Radiographic features of Langerhans’s histiocytosis	4
69.	What is multiple myeloma?	4
70.	Radiographic features of multiple myeloma	4
71.	Location of the jugular foramen	4
72.	Location of the carotid canal	4
73.	Location of foramen rotundum	4
74.	Location of vidian canal	4
75.	Radiographic features of sinusitis	4
76.	Radiographic features of mucositis	4
77.	What is the antral projection?	4
78.	Radiographic appearance of sialolith	4
79.	Radiographic appearance of calcified lymph nodes	4
80.	What is cervical resorption?	4
